# Setting ambitious targets for surveillance and treatment rates among patients with hepatitis C related cirrhosis impacts the cost-effectiveness of hepatocellular cancer surveillance and substantially increases life expectancy: A modeling study

**DOI:** 10.1371/journal.pone.0221614

**Published:** 2019-08-26

**Authors:** Jennifer Uyei, Tamar H. Taddei, David E. Kaplan, Michael Chapko, Elizabeth R. Stevens, R. Scott Braithwaite

**Affiliations:** 1 Division of Comparative Effectiveness and Decision Science, Department of Population Health, New York University School of Medicine, New York, NY, United States of America; 2 VA Connecticut-Healthcare System, West Haven, CT, United States of America; 3 Corporal Michael J. Crescenz VA Medical Center, Philadelphia, PA, United States of America; 4 Center of Innovation for Veteran-Centered and Value-Driven Care, Health Services Research & Development, VA Puget Sound, Seattle, WA, United States of America; University of Texas Medical School at Houston, UNITED STATES

## Abstract

**Background:**

Hepatocelluar cancer (HCC) is the leading cause of death among people with hepatitis C virus (HCV)-related cirrhosis. Our aim was to determine the optimal surveillance frequency for patients with HCV-related compensated cirrhosis.

**Methods:**

We developed a decision analytic Markov model and validated it against data from the Veterans Outcomes and Costs Associated with Liver Disease (VOCAL) study group and published epidemiologic studies. Four strategies of different surveillance intervals were compared: no surveillance and ultrasound surveillance every 12, 6, and 3 months. We estimated lifetime survival, life expectancy, quality adjusted life years (QALY), total costs associated with each strategy, and incremental cost effectiveness ratios. We applied a willingness to pay threshold of $100,000. Analysis was conducted for two scenarios: a scenario reflecting current HCV and HCC surveillance compliance rates and treatment use and an aspirational scenario.

**Results:**

In the current scenario the preferred strategy was 3-month surveillance with an incremental cost-effectiveness ratio (ICER) of $7,159/QALY. In the aspirational scenario, 6-month surveillance was preferred with an ICER of $82,807/QALY because treating more people with HCV led to a lower incidence of HCC. Sensitivity analyses suggested that surveillance every 12 months would suffice in the particular circumstance when patients are very likely to return regularly for testing and when appropriate HCV and HCC treatment is readily available. Compared with the current scenario, the aspirational scenario resulted in a 1.87 year gain in life expectancy for the cohort because of large reductions in decompensated cirrhosis and HCC incidence.

**Conclusions:**

HCC surveillance has good value for money for patients with HCV-related compensated cirrhosis. Investments to improve adherence to surveillance should be made when rates are suboptimal. Surveillance every 12 months will suffice when patients are very likely to return regularly for testing and when appropriate HCV and HCC treatment is readily available.

## Background

Hepatocellular cancer (HCC) is the leading cause of death among people with hepatitis C virus (HCV)-related cirrhosis,[1] with HCV related HCC mortality rapidly increasing from 13 per 100,000 VA patient-years in 2001 to 37 per 100,000 VA patient-years in 2013.[1] HCC incidence in patients with HCV-related cirrhosis is estimated at 1–8% per year,[2–4] and natural history studies report 3-year survival probabilities for untreated patients with small cancers (<5 cm) in the range of 12–21%.[5, 6] Considering the high risk of HCC, guidelines recommend that cirrhotic patients undergo routine ultrasound surveillance at 6–12 month intervals, with or without serial serum alpha-fetoprotein (AFP) testing.[7, 8] The rationale is prompt detection of HCC at earlier stages when more curative treatment options are feasible.[7, 9]

While these guidelines recommend routine HCC surveillance as the standard of care, actual surveillance rates are low. In the United States, the proportion of at-risk patients receiving surveillance, based on pooled data from 9 studies was 18.4% (range, 11–78%), with higher performance in subspecialty gastroenterology clinics compared to primary care clinics (52% vs. 17%).[10] In a recent study of patients enrolled in the United States Department of Veteran Affairs (VA) healthcare system, similar patterns were observed, with 18% (95%CI ± 22%) of at-risk patients receiving HCC surveillance with the strongest predictor of timely surveillance being a visit to a specialist in the first year after cirrhosis diagnosis.[4]

Prior modeling studies have shown that HCC surveillance every 6 months in patients with HCV-related cirrhosis is cost-effective compared to no surveillance (improved survival but at a greater cost), yielding incremental cost effectiveness ratios (ICERs) in the range of $35,300–70,500 per quality-adjusted life-year (QALY).[11–13] In these studies adherence to the recommended surveillance interval is assumed to be 100%, an optimal scenario that is relevant to setting policy on surveillance, but one that may not accurately reflect real-world surveillance performance. Thus, it is unclear if and how the value (i.e., cost per QALY gained) of HCC surveillance changes with varying levels of surveillance adherence. In addition, the value of HCC surveillance has not yet been evaluated in the context of newer direct-acting agents (DAAs), such as sofosbuvir, for treating HCV which have substantially improved cure rates and are cost-effective but expensive.[14–16] Our study aimed to address these knowledge gaps. We developed a decision analytic Markov model that represents the progression of HCV-related cirrhosis, effects of HCV treatment, adherence to HCC ultrasound surveillance, and stage-specific HCC treatments.

## Methods

### Compared strategies

The strategies compared were: 1) no surveillance; 2) ultrasound surveillance every 12 months; 3) ultrasound surveillance every 6 months; and 4) ultrasound surveillance every 3 months. No surveillance was defined as absence of routine HCC surveillance in cirrhotic patients, while allowing for HCV anti-viral treatment, as well as liver transplantation should decompensated cirrhosis develop. The 12, 6, and 3 month surveillance strategies represent different intervals between performing ultrasound surveillance, with the actual occurrence of an ultra sound dependent on adherence to the surveillance schedule.

### Scenarios

Analyses were performed from the perspective of the VA health system. Analysis was carried out for two scenarios. The first scenario reflects current rates of HCV and HCC surveillance and treatment use in the VA health system (HCV treatment coverage was 50%, adherence to HCC surveillance was 20%, and use of curative HCC treatments was 1% for liver transplant, 4% for surgical resection, and 10% for RFA).[4] The second scenario represents aspirational use of HCV and HCC surveillance and treatment (HCV treatment coverage was 100%, adherence to HCC screening was 100%, and use of curative HCC treatments was 34% for liver transplant, 29% for surgical resection, and 20% for RFA). We did not assume 100% use of HCC treatments as other mitigating factors, such as existing liver damage, tumor multifocality, and organ availability (for liver transplant), limits their use in practice. The rate for liver transplant reflects the overall national liver transplant rate of 42 per 100 person-years as reported by the Scientific Registry of Transplant Recipient and Organ Procurement and Transplant Network.[17] The 20% for RFA also reflects an overall national rate as reported by a longitudinal study that includes data from 1044 hospitals in 40 states.[18] Since our search did not uncover national rates for surgical resection, we used an estimate of 29% that reflects the eligibility for liver resection.[19] For each scenario, we projected survival probability, life expectancy, QALY, and costs. To identify the most efficient strategies in each scenario, ICERs for each strategy were compared over a lifetime time horizon. Inefficient strategies were disqualified from consideration based on strong and weak dominance. We used a willingness to pay threshold of $100,000-$150,000 per QALY, which reflects a lower to mid-range estimate for the current US health care system and the recommended range published by the Institute for Clinical and Economic Review.[20] We conducted additional analyses to test if results held true for different aged cohorts (50 and 70 year old when entering the model).

### Cirrhosis disease progression Markov model

**Fig 1:**
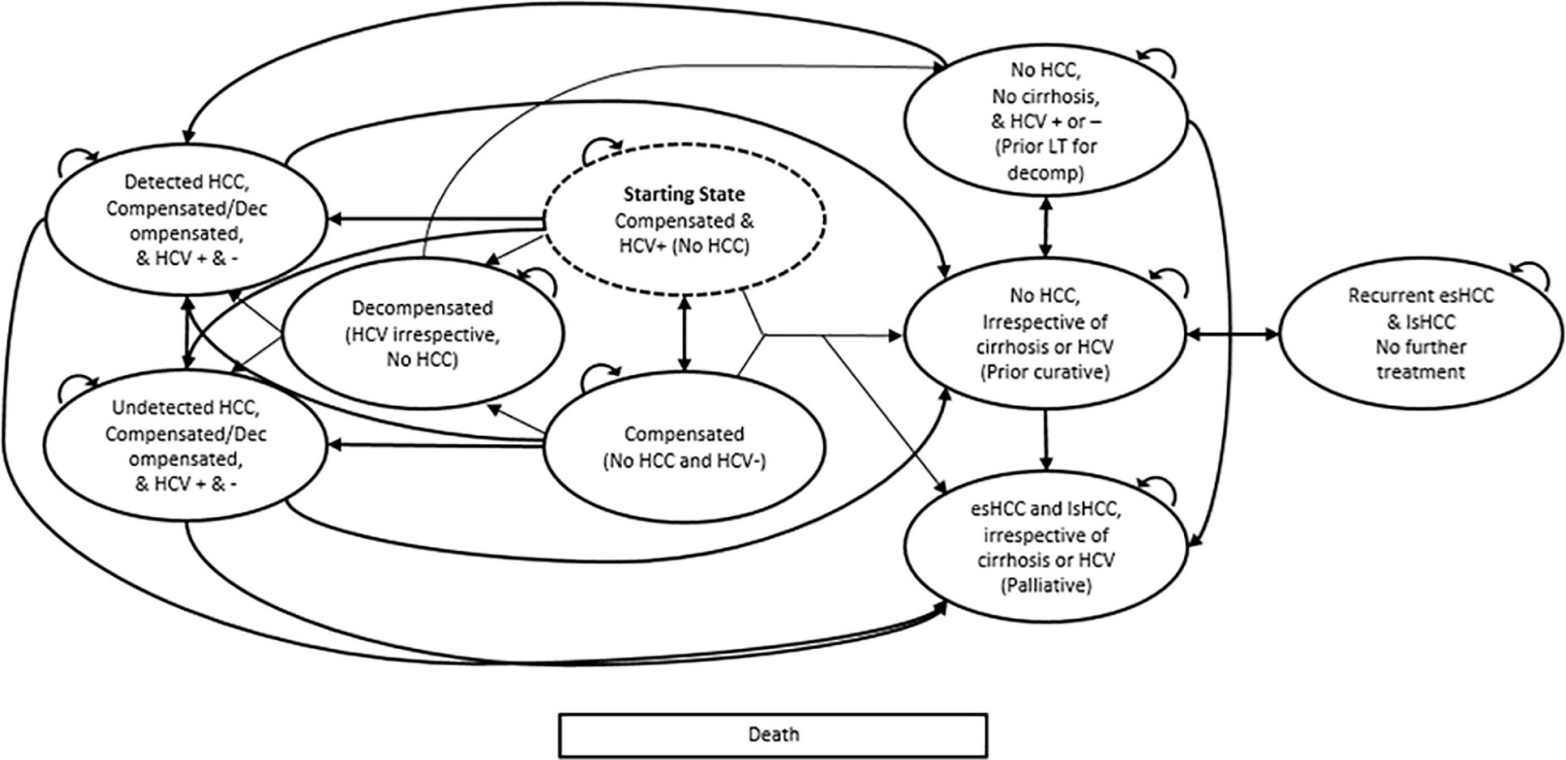
HCV/HCC progression model. esHCC = early stage hepatocellular cancer, HCC = hepatocellular cancer, HCV = hepatitis C virus, lsHCC = late stage hepatocellular cancer. “HCV irrespective” = applies to both HCV-positive and HCV-negative patients. The diagram shows a condensed version of the model. The full model includes 27 mutually exclusive health states based on HCV status, presence of decompensation, cancer stage, detected HCC, HCC cure after treatment, treatment type, cancer recurrence, and death. Ovals represent health states and arrows state transitions. Death is possible in each state.

We developed a decision analytic Markov model simulating progression of cirrhosis to HCC and death among VA patients. The model was used to estimate survival, life expectancy, QALY, total costs associated with each strategy, and ICERs. Fig 1 illustrates the model’s structure, health states, and possible transitions. The initial cohort entered the model at age 60 with chronic HCV, HCV-related compensated cirrhosis, and no HCC, and underwent surveillance according to the assigned strategy. We defined compensated cirrhosis as asymptomatic liver disease and decompensated cirrhosis as the presence of variceal hemorrhage, ascites, encephalopathy, or jaundice.[21, 22] Early stage HCC was defined as Barcelona Clinic Liver Cancer (BCLC) staging classifications 0 and A, and late stage as BCLC stages B-D.[23]

**Fig 2:**
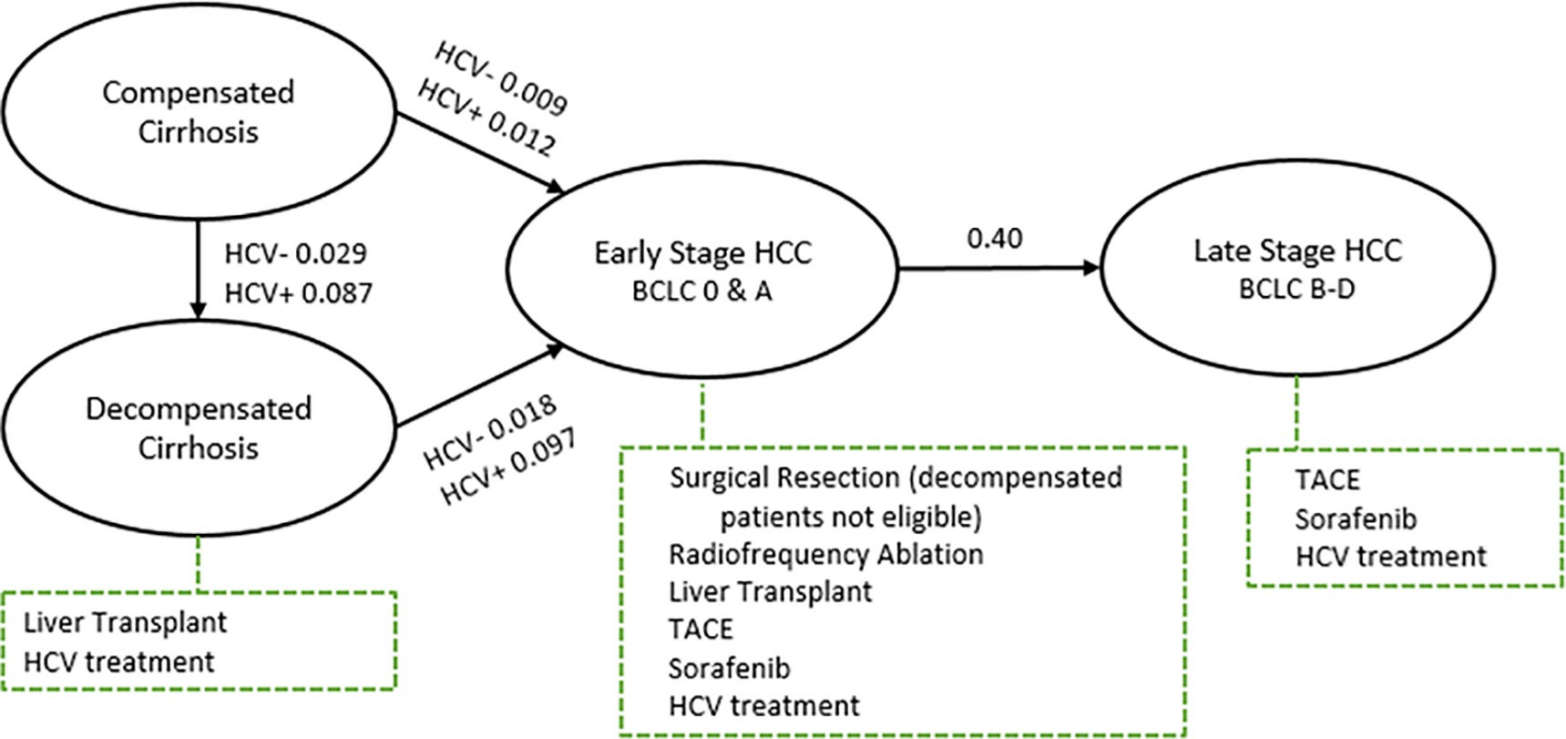
Progression of HCV related compensated cirrhosis and stage-specific treatments. BCLC = Barcelona Clinic Liver Cancer staging classification, HCC = hepatocellular cancer, HCV = hepatitis C virus, and TACE = transarterial chemoembolization. The diagram illustrates how disease progression and stage-specific treatments for HCC are simulated in the model. Numeric values indicate the annual rate of transition with and without HCV.

Fig 2 illustrates the stages and rates of disease progression. Progression from compensated to decompensated cirrhosis for patients cured of HCV infection (i.e., achieving a sustained virologic response (SVR), meaning clearance of HCV viremia 12 weeks or more after completion of HCV antiviral treatment) occurred at an annual rate of 0.3%.[24] The model increased these rates by a factor of 3.0[24] in patients with untreated HCV infection and those who did not achieve SVR. Progression from compensated cirrhosis to early stage HCC occurred at an annual rate of 1%[4, 25] without the presence of HCV and 1.2% with HCV-infection.[4] Among those with decompensated cirrhosis, progression to early stage HCC occurred at an annual rate of 1.8%[4, 25–27] in patients cured of HCV infection and 9.7%[4, 26, 27] in those not treated or cured of HCV infection. Annual HCV antiviral treatment probability was 50% in the current scenario and 100% in the aspirational scenario. Based on a Phase 3 randomized controlled trial of the DAA sofosbuvir to treat HCV, the SVR rate was 92% among patients without cirrhosis and 80% among patients with cirrhosis after 12 weeks of treatment.[28] Because current DAAs report a higher SVR rate of 95–100%,[29] in sensitivity analyses we increased the SVR rate up to 0.95 for both cirrhotic and non-cirrhotic patients.

**Fig 3:**
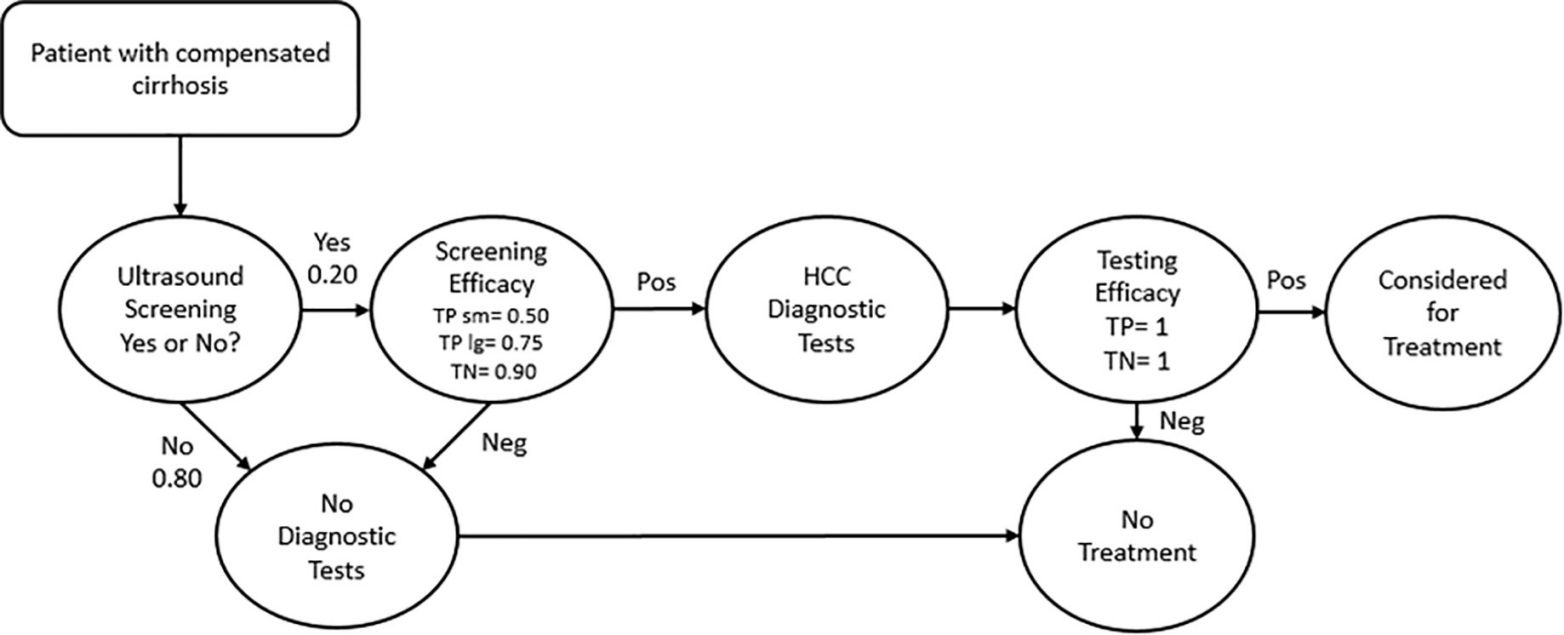
Surveillance, testing, and treatment consideration process modeled. HCC = hepatocellular cancer, TP sm = True positive for small tumors, TP lg = True positive for large tumors, TN = true negative. Those who receive a negative HCC test result re-enter surveillance.

Fig 3 shows the sequence from ultrasound surveillance to diagnostic evaluation for HCC to HCC treatment. The sensitivity of ultrasound surveillance was set at 0.50 for small tumors (< or = 5cm) and 0.75 for large tumors (> 5cm), while specificity was 0.96.[30] Eighty percent of patients with a new liver lesion detected on ultrasound surveillance then underwent diagnostic evaluation for HCC,[31] and followed the AASLD diagnostic algorithm which includes magnetic resonance imaging and/or computed tomography,[7] and if necessary biopsy. For simplicity, we assumed that the diagnostic algorithm used had perfect discriminatory properties, with a confirmed HCC diagnosis leading to consideration for treatment, while a negative or indeterminate result would lead to no HCC treatment.

HCC stage-specific treatments were based on the AASLD practice guidelines (Fig 2).[32] While we designed our analyses to be relevant across diverse settings and health care systems, we analyzed VA operational data to estimate the likelihood a patient would receive a treatment given s/he was eligible in the current scenario.[4] Based on that data, the annual likelihood of liver transplant was 1%, 4% for surgical resection, and 10% for RFA. For the aspirational scenario, we did not assume 100% treatment use as other mitigating factors, such as existing liver damage, tumor multifocality, and organ availability (for liver transplant), limits their use in practice. Instead estimates reflect either eligibility rates or national treatment utilization rates. The annual probability for liver transplant was 20%, 15% for surgical resection, and 30% for RFA. We assumed surgical treatments were successful in removing the cancer with an annual recurrence rate of 26.1%[33] for surgical resection, 16.3%[34] for RFA, and 5.9%[35] for liver transplantation. With recurrence, additional treatments were available and the likelihood of treatment followed the same stage-specific probabilities as described earlier.

To account for harms associated with treatments we applied a one-time likelihood of major complications and death attributable to each surgical procedure that occurred within the first 30 days post-surgery. Based on the literature, for surgical resection 9.5% of patients experienced a major complication and 2.4% died from procedure-related causes;[36] for RFA, 2.4% of patients experienced a major complication and 0.26% died;[37] and for liver transplantation 38.7% of patients had major complications and 1.9% died.[38] As palliative treatments for HCC are administered over a longer period of time than curative treatments, we applied an annual rate of 14.3%[39] for adverse events in patients undergoing TACE and 42.1%[40] for patients receiving sorafenib over the duration of the specified treatment. An annual mortality rate due to treatment of 1.9%[39] was assumed for both palliative treatments.

Mortality was calculated based on age (United States life tables[41]), disease status, and treatment (Table 1). TreeAge Pro and Microsoft Excel were used to develop the model.[42]

**Table 1:** Annual probability of excess mortality by disease and treatment.

		No HCC	Early stage HCC	Late stage HCC
Compensated cirrhosis				
	No treatment	0.05[43]	0.41[5]	0.67[40]
	Surgical resection	NA	0.11[34]	—^a^
	Radiofrequency ablation	NA	0.10[34]	—^a^
	Liver transplant	NA	0.04[17]	0.04[17]
	Sorafenib	NA	0.32[40]	0.32[40]
	TACE	NA	0.17[39]	0.17[39]
Decompensated cirrhosis				
	No treatment	0.38[43]	0.41[5]	0.67[40]
	Surgical resection	NA	—^a^	—^a^
	Radiofrequency ablation	NA	—^a^	—^a^
	Liver transplant	0.04[17]	0.04[17]	0.04[17]
	Sorafenib	NA	0.32[40]	0.32[40]
	TACE	NA	0.17[39]	0.17[39]

^a^ = specified treatment not recommended in the AASLD practice guidelines for that particular cancer stage

HCC = hepatocellular cancer, NA = not applicable, TACE = transarterial chemoembolization.

NOTE: Excess mortality is the added risk of dying due to a disease, with or without treatment. The excess risk is added to the age-specific risk based on United States life tables to derive a total risk of death. In calculating excess risk, age-specific mortality of the study cohort was removed in order to isolate risk attributable to the disease. Additionally, values from the literature have been converted from their original state (i.e. multi-year rates, RR etc.) to annual probabilities.

### Model validation

We validated the model by comparing model-generated HCC incidence rates and survival curves to data from the Veterans Outcomes and Costs Associated with Liver Disease (VOCAL) study group[4] and published epidemiologic studies. Adequate goodness of fit was demonstrated for all targets. To compare estimates to VOCAL data we simulated a similar cohort of patients who entered the model at 60 years of age, of whom 70% had compensated cirrhosis, 30% had decompensated cirrhosis, and none were treated for HCV. Annual HCC incidence for the simulated cohort was 0.98%, which was similar to the VOCAL estimate of 1%. Survival at 5 years for the simulated cohort was 55.6%, compared to 56.3% for the VOCAL study group [S1 Fig]. We also simulated a scenario in which HCC surveillance and treatment were not performed to compare simulated survival curves to epidemiologic studies reporting on the natural history of compensated cirrhosis. Survival at 5 years for the simulated cohort was 77.4%, in agreement with the published range of 76.7%-84% (mean deviation of 4.3%)[S2 Fig].[24, 44–46] For liver transplant patients, the model projected 5, 8 and 14-year life expectancy was 70.8%, 55.8%, and 34.5% which also was similar to published data 67.8–83%,[47–49] 55%,[47] and 30%[48] respectively. Based on these results, no additional model adjustment was employed.

### Sensitivity and threshold analyses

Sensitivity analysis tests the degree to which the model’s outcomes are affected by changes in data inputs across plausible ranges. We performed one-way sensitivity analyses for each input value across its plausible range to test the effect of the changes on the model’s outcomes. In addition, we performed threshold analyses for variables identified in one-way sensitivity analyses as having large effect on downstream costs and/or utility, in order to identify the thresholds at which estimates for these variables would change the model’s key inferences for decision making, even if their plausibility was low.

### Model parameters, costs, and utilities

Model input parameters were derived from VA data and published data (Table 2). Each health state was assigned a health utility value from the literature. When a utility value was unavailable for health states with comorbidities (e.g. cirrhosis and HCC) the condition with the lowest utility value was selected to represent that health state.

**Table 2:** Model inputs.

Variable		Base case (Plausible range)	Reference
Ultrasound surveillance adherence			
	Aspirational scenario	1 (0–1)	
	Current scenario	0.20 (0.10–1)	[4]
*Disease progression*			
Compensated to decompensated cirrhosis			
	HCV-negative	0.0031 (0.001–0.01)	[24]
	HCV-positive, relative risk	11.1 (1.10–8.70)	[24]
Compensated to early stage HCC			
	HCV-negative	0.01 (0.01–0.02)	[4, 25]
	Hazard rate HCV-negative vs. HCV-positive	0.23 (0.03–0.04)	[4]
	HCV-positive	0.01 (0.01–0.02)	[4]
Decompensated to early stage HCC			
	HCV-negative	0.02 (0.08–0.14)	[4, 25–27]
	HCV-positive	0.10 (0.07–0.18)	[4]
	Hazard rate decompensated vs. compensated	8.08 (2.20–29.65)	[26, 27]
Early stage to late stage HCC		0.40 (0.30–0.60)	[5]
*HCV treatment*			
Probability of treatment			
	Current scenario	1 (0–1)	Assumption
	Aspirational scenario	0.50 (0–1)	Assumption
Probability of SVR among patients with cirrhosis after 12 weeks of treatment		0.80 (0.67–0.95)	[15, 28]
Probability of SVR among patients without cirrhosis after 12 weeks of treatment		0.92 (0.89–0.95)	[28]
*Ultrasound surveillance and diagnostic tests*			
Ultrasound surveillance			
	Sensitivity for small tumors	0.50 (0.17–0.62)	[30, 50, 51]
	Sensitivity for large tumors	0.75 (0.75–0.94)	[30, 50]
	Specificity	0.96 (0.92–0.96)	[30, 50, 51]
Probability of diagnostic testing after positive ultrasound result		0.80	[31]
*HCC stage specific treatments*			
*Annual probability of treatment*			
Surgical resection			
	Current scenario	0.04 (0.03–0.29)	VA operational data
	Aspirational scenario	0.15 (0.03–0.29)	Assumption
Radiofrequency ablation			
	Current scenario	0.10 (0.07–0.20)	VA operational data
	Aspirational scenario	0.30 (0.07–0.30)	[18]
Liver transplant			
	Current scenario	0.01 (0.01–0.34)	VA operational data
	Aspirational scenario	0.20 (0.01–0.34)	Assumption
Sorafenib		0.29 (0.22–0.37)	VA operational data
TACE		0.23 (0.18–0.29)	VA operational data
*Probability of adverse events*			
Surgical resection		0.10 (0.07–0.33)	[36, 52]
Radiofrequency ablation		0.02 (0.02–0.10)	[37, 52]
Liver transplant		0.39 (0.29–0.48)	[38]
Sorafenib		0.42 (0.32–0.53)	[39]
TACE		0.14 (0.11–0.18)	[39]
Probability of death attributable to the procedure			
Surgical resection		0.02 (0.01–0.04)	[36, 53]
Radiofrequency ablation		0.003 (0.002–0.004)	[37, 52]
Liver transplant		0.02 (0.01–0.13)	[38, 54]
Sorafenib		0.02 (0.01–0.024)	[39]
TACE		0.02 (0.01–0.024)	[39]
HCC Recurrence after specific treatment			
Surgical resection		0.261 (0.17–0.36)	[13, 33, 55]
Radiofrequency ablation		0.163 (0.12–0.31)	[34, 56]
Liver transplant		0.059 (0.04–0.27)	[35, 57, 58]
*Costs (2016 U*.*S*.*$)*			
Ultrasound HCC surveillance*		129	[59]
Biopsy*		726	[59]
Computerized tomography*		269	[59]
Magnetic resonance imaging*		472	[59]
HCV antiviral (per regimen)		15,000–30,000	[60], personal communication
Liver transplant		86,958	Data from VA HERC via personal communication
Liver transplant complications		66,064	Data from VA HERC via personal communication
Radiofrequency ablation		2,598	[59]
Radiofrequency ablation complications		202	Assumption estimated as 10% of surgical resection complication cost
Sorafenib (annual)		11,629	[60], Data from VA HERC via personal communication
Sorafenib complications		101	Assumption estimated as 5% of surgical resection complication cost
Surgical resection		46,435	Data from VA HERC via personal communication
Surgical resection complications		3,206	Data from VA HERC via personal communication
TACE (annual)		5,661	[59]
TACE complications		403	Assumption estimated as 20% of surgical resection complication cost
Terminal care, death related to surgery		19,550	[61], personal communication with VA staff
Terminal care, related to HCC or liver disease		27,094	[62]
Utilities			
HCV no SVR		0.75 (0.55–0.95)	[63]
HCV yes SVR		1 (0.8–1)	[64], Assumption
Compensated cirrhosis		0.71 (0.57–0.91)	[63]
Decompensated cirrhosis		0.57 (0.37–0.71)	[63]
Early stage HCC		0.61 (0.41–0.81)	[65]
Late stage HCC		0.31 (0.11–0.51)	[66]

*Include costs associated with ordering and communication test results.

HCC = hepatocellular cancer, HCV = hepatitis C virus, SVR = sustained virologic response, and TACE = transarterial chemoembolization.

NOTE: All rates and probabilities are annual

The cost data were primarily obtained from the VA Health Economics Resource Center (HERC), adhering with VA information security policies and the US Centers for Medicare and Medicaid Services (CMS). A cost-to-charge ratio developed by HERC was applied to CMS data (a scaling factor of 1.05 for diagnostic services, 1.20 for inpatient care, and 1.59 for medical services) to estimate VA costs. For diagnostic tests, as we could not determine the actual proportion of patients who received computerized tomography (CT), magnetic resonance imaging (MRI), and/or biopsy (singularly or in combination), we assumed that both CT and MRI were used with a 10% likelihood of biopsy. Since costs for RFA complications were not differentiated like for surgical resection and liver transplantation, we assumed they were equivalent to 10% of surgical resection complication costs. Similarly, costs of complications related to sorafenib and TACE, were derived from surgical resection complication costs, with complication costs set at 5% and 20% (respectively) of those for resections. The cost of surgical deaths was derived from published data on end-of-life care costs,[61] as was the cost of death related to cancer or decompensated cirrhosis.[62] All costs were converted into 2016 dollars using the US Consumer Price Index[67] and costs were discounted at an annual rate of 3%. Effectiveness was also discounted at an annual rate of 3%.

## Results

### Current scenario

**Fig 4:**
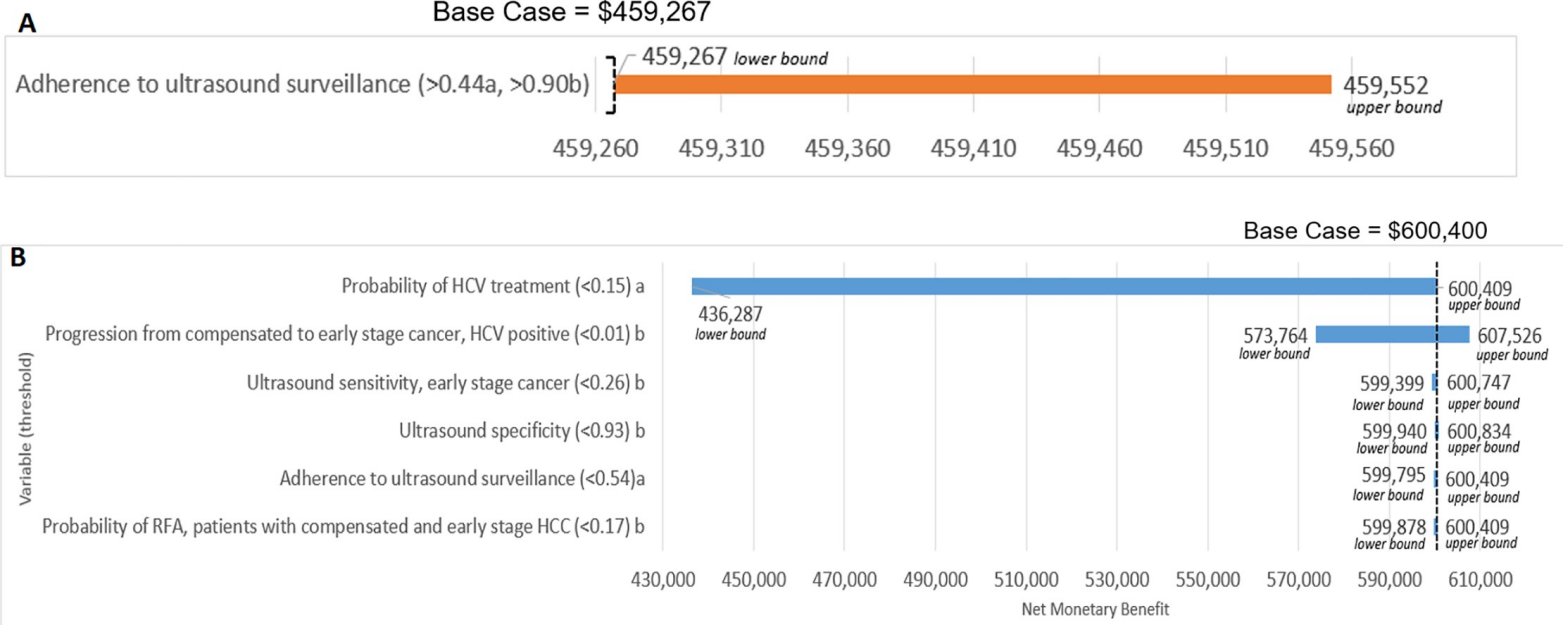
**One-Way Sensitivity Results for the (a) Current Scenario and the (b) Aspirational Scenario. A.** Results were sensitive to variation in adherence to ultrasound surveillance. Net monetary benefit is shown for the lower and upper bounds of the plausible range tested in sensitivity analysis. Thresholds for when the preference switched away from 3-month surveillance are shown in parentheses. Legend: a. When adherence was between 0.44–0.89 the preferred strategy was 6-month surveillance, b. When adherence was 0.90 or greater the preferred strategy was 12-months surveillance. **B.** Results were sensitive to variation for the variables shown in the figure. Net monetary benefit is shown for the lower and upper bounds of the plausible range tested in sensitivity analysis. Thresholds for when the preference switched away from 6-month screening are shown in parentheses. Legend: a. Below the threshold the preferred strategy was 3 months, b. Below the threshold the preferred strategy was 12 months.

Under the current scenario, the preferred strategy was 3-month surveillance followed by 6-month surveillance (ICER = $7,159/QALY; see Table 3). Both 12-month surveillance and no surveillance were dominated. Differences in effectiveness and cost between strategies were minimal. Compared to no surveillance, 3-month surveillance resulted in a 16.44 year increase in life expectancy ($180,000 decrease in cost) per 1,000 people screened, a 10.96 year increase for 6-month surveillance ($204,000 decrease in cost), and a 5.48 year increase for 12-month surveillance ($134,000 decrease in cost). One-way sensitivity analysis showed results were sensitive to variation in the level of adherence to surveillance (Fig 4A). When adherence levels were between 44–90% 6-month surveillance was preferred and at levels above 90% 12-month surveillance was preferred. Results were insensitive to the age of the entering cohort (50 and 70 years old). Varying other inputs within their plausible ranges did not created a change in preferred surveillance frequency.

**Table 3:** Most efficient strategies for current and aspirational scenarios.

Surveillance strategy	LY	QALY	Cost, $	ICER (Δ Cost/Δ QALY), $
*Current scenario*				
No Screening	7.087	4.956	37,258	Dominated ^a^
12 months	7.092	4.958	37,125	Dominated ^a^
6 months	7.097	4.960	37,054	—
3 months	7.104	4.963	37,078	7,159
*Aspirational scenario*				
No Screening	8.922	6.470	47,066	Dominated ^a^
12 months	8.950	6.472	46,898	—
6 months	8.964	6.480	47,576	82,807
3 months	8.977	6.489	49,756	246,245

^a^ This strategy is less effective and more expensive than an alternative and has been eliminated from further consideration.

### Aspirational scenario

**Fig 5:**
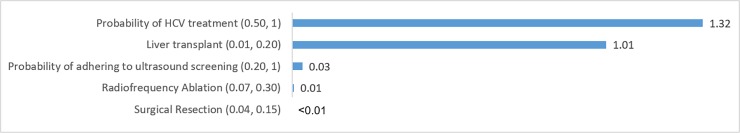
Life expectancy gains when key variables are raised to their aspirational value, one-way sensitivity analysis results. Graph shows gains in life expectancy (years) when variables were increased from their base case value to their aspirational value. Base case and aspirational values are in parentheses.

Under the aspirational scenario, the preferred strategy was 6-month surveillance (ICER = $82,807/QALY compared to 12-month surveillance). Three-month surveillance, compared to 6-month surveillance, was more effective (difference of 0.01 QALY) and more expensive (difference of $2,180) but the strategy was considered inefficient at a WTP threshold of $100,000-$150,000 (ICER = $246,245/QALY). The no screening strategy was dominated (i.e., less effective and more costly than an alternative). Surveilling at 6-month intervals under the aspirational scenario improved life expectancy by 41.1 year increase in life expectancy per 1,000 people screened compared with no surveillance. The 6-month surveillance strategy in the aspirational scenario provided 8.96 LY compared to the current adherence scenario which provided a total of 7.097 LY, representing a 1.87 year gain in life expectancy (see Table 3). One-way sensitivity analysis showed that the preferred strategy switched from 6 to 12-month surveillance when progression from compensated cirrhosis to early stage cancer for those with HCV was below 0.01, ultrasounds sensitivity for early stage cancer was below 0.26, ultrasound specificity fell below 0.93, or probability of RFA was below 0.17. The preferred strategy switched from 6 to 3-month surveillance when the likelihood of HCV treatment was below 0.15 or adherence to ultrasound surveillance was under 0.54. (Figs 4B & 5) Varying other inputs within their plausible ranges (Table 2) did not created a change in preferred surveillance frequency. While sensitivity analyses were performed for all variables, only those with significant results are presented.

## Discussion

The results of our study suggest that the currently recommended 6-month surveillance interval for patients with HCV-related compensated cirrhosis has good value for money under an aspirational scenario but the strategy loses value when adherence to surveillance is low. Under the current scenario (low adherence), 3-month surveillance was preferred in an effort to survey as many patients, but 6-month surveillance was preferred once adherence levels reached 44–90%, and 12-month surveillance was preferred once adherence reached 90% or greater. These findings are in line with conclusions in support of 6-month HCC surveillance drawn from a recent large randomized trial that showed increasing surveillance intervals from 6-month to 3-months, under circumstances of adequate compliance and with examinations occurring as scheduled, did not improve health outcomes.[68] Policy and operational procedures should be designed to ensure adequate levels of surveillance adherence among this defined population and to invest in strategies that will increase surveillance frequency among patient groups with suboptimal adherence.

While HCC screening is important for this population, the analysis also shows that significant gains in life expectancy are achieved with improved use of key services. Comparing the 6-month surveillance strategy under the two scenarios (current versus aspirational), an added 1.87 life years was projected for the cohort (see Table 3). To examine the impact each variable had on life expectancy, we conducted one-way sensitivity analysis using the current scenario and increased each key variable independently to their aspirational value (see Fig 5). Much of the gain is attributable to the increase in liver transplant probability among patients with decompensated cirrhosis who have not yet developed HCC (data not shown), and increase in HCV treatment coverage which slowed the progression to developing HCC. This suggests that for this particular patient population, interventions that help prevent HCC and decompensated cirrhosis have the greatest impact on life expectancy. With a reduction in DAA prices due to a more competitive market, it is possible that greater HCV treatment coverage can be realized. By decreasing the incidence of HCC associated with HCV, increased DAA coverage has the potential to make the cost effectiveness of more frequently screening less favorable. However, for those who do develop HCC, early detection and use of surgical intervention is important to improve life expectancy. Comparing life expectancy of patients with detected (via surveillance) versus undetected cancer among patients with early stage HCC, we estimate an average 0.85 year gain in life expectancy per patient and an average cost saving of $40,720 per patient under the aspirational scenario.

Our model’s predicted effectiveness (expected life years and QALYs) is similar to that obtained in other published models of HCC surveillance (6.8 to 8.6 expected life years and 5.3 to 6.3 QALYs for no surveillance, and 7.1 to 9.1 expected life years and 5.5 to 6.7 QALYs for 6-month surveillance).[11, 13] Our predicted total costs are lower, likely since they reflect VA costs which are substantially discounted compared to non-VA health care organizations. The lower costs in our study likely explain the discrepancy in our cost-effectiveness results compared to published studies. While our model found surveillance to be cost-saving compared with no surveillance (more effective and less expensive), other published models of HCC surveillance have reported ICERs in the range of $35,292-$70,494,[11–13] suggesting that surveillance is more effective but more expensive under the assumptions used in those models.

Our study has several notable limitations. First, the analyses used a VA health system perspective and used VA specific inputs that may not be applicable to other health systems. Second, in the aspirational scenario we acknowledge that certain clinical factors could be more prevalent in the VA population that prohibits greater use of surgical treatments, and that organ availability may limit liver transplantation. Thus, it is possible that the treatment rates used in the aspirational scenario are unrealistic. However substantial improvement is possible as it is recognized that HCC-specific treatment rates in the VA are low and variation exists based on whether the patient was surveilled and if the patient was seen by a surgeon or oncologist.[69] Third, in order to capture the natural history of HCV and avoid confounding from treatment, we relied on studies that occurred prior to treatment. Although, the inputs were derived from older studies, the natural history of untreated HCV infection is not likely to have changed over time, sensitivity analyses demonstrated that this assumption is unlikely to impact model outcomes. Fourth, our model applies HCC surveillance adherence rates uniformly across the cohort over time (all patients have the same adherence rate) without considering that in actuality, some patients adhere more than others and some patients possibly will never commit to regular or consistent surveillance. Similarly, some providers order surveillance studies inconsistently or not all; this factor may be mitigated by use of electronic clinical reminders and other decision support tools.[70] This likely explains why more frequent surveillance is favored when adherence levels are low, as the model will apply the likelihood of surveillance equally across a cohort without differentiating between adherent v. non-adherent patients. Therefore, among adherent patients less frequent screenings, as seen in the aspirational scenario may be more applicable and less adherent patients may require more reminders and screenings scheduled to achieve similar outcomes. Additionally, as data was not available for some inputs, such as Sorafenib and TACE treatment effects for all subgroups, the treatment effects were assumed to be equivalent across subgroups, which is unlikely to be true in practice. Moreover, assumptions were made for some inputs such as assuming the HSUV for those achieving SVR was that of a healthy individual, which may overestimate the impact of curing HCV. Sensitivity analyses demonstrated that these assumptions are unlikely to significantly impact model outcomes. Fifth, health utility values were selected from a range of assessment tools when results from the designated reference tool (i.e. SF-6D) were unavailable. While this is standard practice it can be problematic to combine HSUV estimates calculated using different instruments, since those values are typically not equivalent for the same health states. To address this issue sensitivity analyses were performed that indicated that the variation in utility values was unlikely to impact the outcome of the analysis. Finally, given the highly complex nature of HCV disease progression to HCC, as well as the intricacies of the system of health care delivery, we made a number of simplifying assumptions in order to contain the size of the model. For example, we do not model comorbidities such as alcohol use disorder or NASH, which could potentially alter the rate of disease progression for a proportion of patients, adherence to HCC surveillance and treatment, and the effectiveness of HCC treatments. Despite these simplifications, we believe the model presented here captures the most salient characteristics of disease progression and health care utilization in this specified population and adds details not found in other models, including HCC stage-specific treatments and HCC recurrence.

## Conclusion

In summary, routine HCC surveillance has good value for money for patients with HCV related compensated cirrhosis and investments to improve adherence to surveillance should be made when rates are suboptimal. Surveillance every 12 months will suffice in the particular circumstance when patients are very likely to return regularly for testing and when appropriate HCC treatment is readily available. When patient compliance and HCC treatment are not optimal, surveillance every 3 to 6 months is recommended. For this patient population, interventions that slowed or prevented HCC from developing had the greatest impact on life expectancy.

## Supporting information

S1 Fig Model validation: Model predicted 5-year surival compared to VOCAL screening. (DOCX)

S2 Fig Model predicted HCC natural history 5-year survival vs. epidemiological studies. (DOCX)

S1 Table Rate and probability model inputs and their conversion to 1 year values. (DOCX)

## Abbreviations

HCC: hepatocellular cancer
HCV: hepatitis C
ICER: incremental cost effectiveness ratios
QALY: quality adjusted life years
RFA: radiofrequency ablation
VA: United States Department of Veterans Affairs
VOCAL: Veterans Outcomes and Costs Associated with Liver Disease
