# Supplementary figures and images for: Setting ambitious targets for surveillance and treatment rates among patients with hepatitis C related cirrhosis impacts the cost-effectiveness of hepatocellular cancer surveillance and substantially increases life expectancy: A modeling study
Source: PLoS One. 2019 Aug 26;14(8):e0221614. doi: 10.1371/journal.pone.0221614 (PMC6709904; doi:10.1371/journal.pone.0221614)

S1 Figure. Model Validation: Model Predicted 5-year surival compared to VOCAL screening


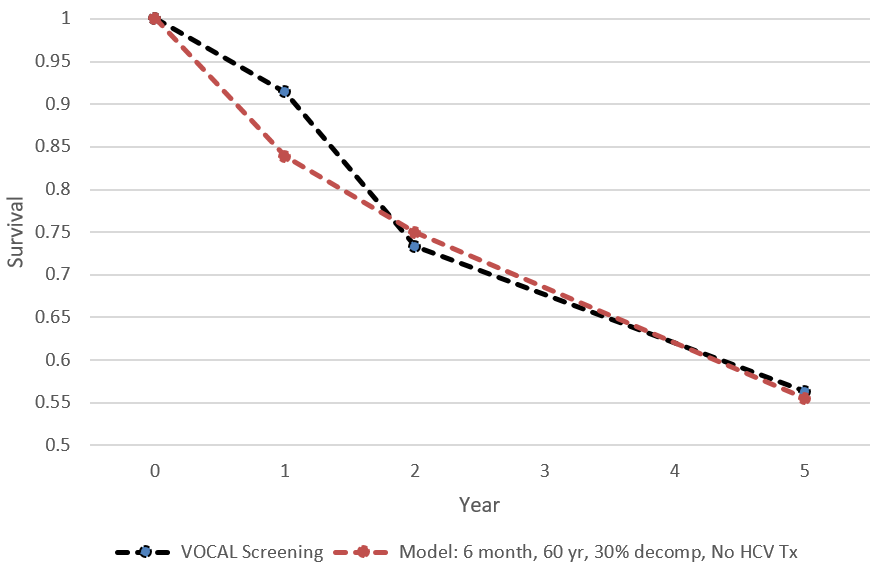

Supplement: S1 Fig — (DOCX) [file pone.0221614.s001.docx]

S2 Figure. Model predicted HCC natural history 5-year survival vs. epidemiological studies


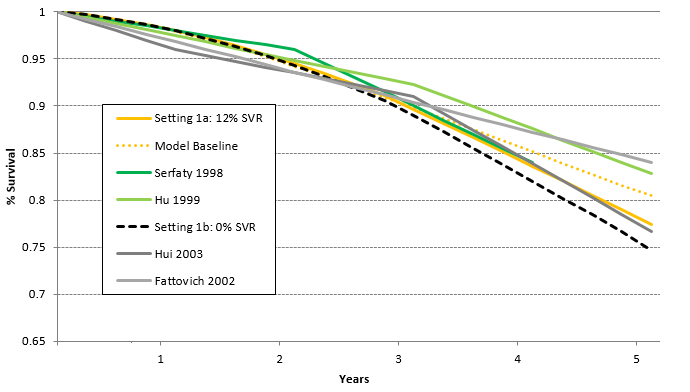

Supplement: S2 Fig — (DOCX) [file pone.0221614.s002.docx]
