# Supplementary material for: Setting ambitious targets for surveillance and treatment rates among patients with hepatitis C related cirrhosis impacts the cost-effectiveness of hepatocellular cancer surveillance and substantially increases life expectancy: A modeling study
Source: PLoS One. 2019 Aug 26;14(8):e0221614. doi: 10.1371/journal.pone.0221614 (PMC6709904; doi:10.1371/journal.pone.0221614)
Supplement: S1 Table — (DOCX) [file pone.0221614.s003.docx]

S1 Table. Rate and probability model inputs and their conversion to 1 year values

| Variable | Base case (Plausible range) | Reference |
| --- | --- | --- |
| Ultrasound surveillance adherence  Ideal scenario  Current scenario | 1 (0-1)  0.20 (0.10-1) | [^4^](#_ENREF_4) |
| *Disease progression* |  |  |
| Compensated to decompensated cirrhosis  HCV-negative  HCV-positive, relative risk | 0.0031 (0.001-0.01)  11.1 (1.10-8.70) | [^24^](#_ENREF_24)  [^24^](#_ENREF_24) |
| Compensated to early stage HCC  HCV-negative  Hazard rate HCV-negative vs. HCV-positive  HCV-positive | 0.01 (0.01-0.02)  0.23 (0.03-0.04)  0.01 (0.01-0.02) | [^4^](#_ENREF_4)^,^[^25^](#_ENREF_25)  [^6^](#_ENREF_6)  [^4^](#_ENREF_4) |
| Decompensated to early stage HCC  HCV-negative  HCV-positive  Hazard rate decompensated vs. compensated | 0.02 (0.08-0.14)  0.10 (0.07-0.18)  8.08 (2.20-29.65) | [^4^](#_ENREF_4)^,^[^25-27^](#_ENREF_25)  [^4^](#_ENREF_4)  [^26^](#_ENREF_26)^,^[^27^](#_ENREF_27) |
| Early stage to late stage HCC | 0.40 (0.30-0.60) | [^5^](#_ENREF_5) |
| *HCV treatment* |  |  |
| Probability of treatment  Current scenario  Aspirational scenario | 0.50 (0-1)  1 (0-1) | ^Assumption^ |
| Probability of SVR among patients with cirrhosis after 12 weeks of treatment | 0.80 (0.67-0.95) | [^15^](#_ENREF_15)^,^[^28^](#_ENREF_28) |
| Probability of SVR among patients without cirrhosis after 12 weeks of treatment | 0.92 (0.89-0.95) | [^28^](#_ENREF_28) |
| *Ultrasound surveillance and diagnostic tests* |  |  |
| Ultrasound surveillance |  |  |
| Sensitivity for small tumors | 0.50 (0.17-0.62) | [^30^](#_ENREF_30)^,^[^55^](#_ENREF_55)^,^[^56^](#_ENREF_56) |
| Sensitivity for large tumors | 0.75 (0.75-0.94) | [^30^](#_ENREF_30)^,^[^55^](#_ENREF_55) |
| Specificity | 0.96 (0.92-0.96) | [^30^](#_ENREF_30)^,^[^55^](#_ENREF_55)^,^[^56^](#_ENREF_56) |
| Probability of diagnostic testing after positive ultrasound result | 0.80 | [^31^](#_ENREF_31) |
| *HCC stage specific treatments* |  |  |
| Annual probability of treatment |  |  |
| Surgical resection  Current scenario  Aspirational scenario | 0.04 (0.03-0.29)  0.15 (0.03-0.29) | ^VA operational data^  ^Assumption^ |
| Radiofrequency ablation  Current scenario  Aspirational scenario | 0.10 (0.07-0.20)-  0.30 (0.07-0.30) | ^VA operational data^  [^18^](#_ENREF_18) |
| Liver transplant  Current scenario  Aspirational scenario | 0.01 (0.01-0.34)  0.20 (0.01-0.34) | ^VA operational data^  ^Assumption^ |
| Sorafenib | 0.29 (0.22-0.37) | ^VA operational data^ |
| TACE | 0.23 (0.18-0.29) | ^VA operational data^ |
| Probability of adverse events |  |  |
| Surgical resection | 0.10 (0.07-0.33) | [^36^](#_ENREF_36)^,^[^57^](#_ENREF_57) |
| Radiofrequency ablation | 0.02 (0.02-0.10) | [^37^](#_ENREF_37)^,^[^57^](#_ENREF_57) |
| Liver transplant | 0.39 (0.29-0.48) | [^38^](#_ENREF_38) |
| Sorafenib | 0.42 (0.32-0.53) | [^20^](#_ENREF_20) |
| TACE | 0.14 (0.11-0.18) | [^39^](#_ENREF_39) |
| Probability of death attributable to the procedure |  |  |
| Surgical resection | 0.02 (0.01-0.04) | [^36^](#_ENREF_36)^,^[^58^](#_ENREF_58) |
| Radiofrequency ablation | 0.003 (0.002-0.004) | [^37^](#_ENREF_37)^,^[^57^](#_ENREF_57) |
| Liver transplant | 0.02 (0.01-0.13) | [^38^](#_ENREF_38)^,^[^59^](#_ENREF_59) |
| Sorafenib | 0.02 (0.01-0.024) | [^39^](#_ENREF_39) |
| TACE | 0.02 (0.01-0.024) | [^39^](#_ENREF_39) |
| HCC Recurrence after specific treatment |  |  |
| Surgical resection | 0.261 (0.17-0.36) | [^13^](#_ENREF_13)^,^[^33^](#_ENREF_33)^,^[^60^](#_ENREF_60) |
| Radiofrequency ablation | 0.163 (0.12-0.31) | [^34^](#_ENREF_34)^,^[^61^](#_ENREF_61) |
| Liver transplant | 0.059 (0.04-0.27) | [^35^](#_ENREF_35)^,^[^62^](#_ENREF_62)^,^[^63^](#_ENREF_63) |
